# Creating learner-centered assessment strategies for promoting greater student retention and class participation

**DOI:** 10.3389/fpsyg.2014.00595

**Published:** 2014-06-19

**Authors:** John D. Rich, Arabia N. Colon, Dominique Mines, Kimberly L. Jivers

**Affiliations:** Psychology, Delaware State UniversityDover, DE, USA

**Keywords:** assessment, learner-centered, engagement, higher-order cognition, psychology

Many teachers still use in-class multiple choice exams in their classes, the primary goal of which is to see how much the students have already learned. The assessment strategies we will examine in this paper change the focus from assessing *whether* students have learned anything to creating assessments which double as learning experiences themselves. Assessments do not have to merely measure *what* was learned; rather, they can be methods for getting students to learn while they are completing the task you have given them. The theoretical framework of learner-centered assessment emphasizes problem solving, higher order thinking skills, the promotion of a sense of ownership in learning, and a dialogic approach to instruction (Rich, 2011).

The purpose of this paper is to discuss six specific strategies for implementing learner-centered assessment in the classroom.

The six research based strategies we will discuss are:

- Strategies which ensure students have read the material
- The use of take-home examinations
- Giving short answer tests with questions at an integrative and/or applied level on Bloom's taxonomy
- Using Formative summative assessments during class time (FSA)
- Being responsive to results from Audience response systems (ARS)
- Student learning style inventories

The students have to read the material to learn anything from it!

A key to effective teaching is to ensure that students have read all the material. If the student doesn't read the material they will not be as ready to understand what is going on when the teacher covers the work in class (Krashen, 2004). As an instructor, breaking up the material may be beneficial because many students just breeze through the chapter instead of actually reading it. If teachers ask a question about the assigned reading at the beginning of the first class when that information will be discussed, and students are informed that there will be an in-class quiz on the reading, more students will do the reading (Sweet et al., 1998). If your students persist on ignoring their assigned readings, according to Felder and Silverman (1988) and Lucas and Bernstein (2004), there is not much point in punishing them. The students who fail to read will then be punished enough on examinations and quizzes.

## Take-home exams

Despite the common perception that take-home examinations are “giveaways” by teachers with low expectations, some research (e.g., Rich, 2011) demonstrates that the process of preparing a submission for a take-home test, can produce longer retention of material than studying for in-class examinations. When a student is answering items on a take-home exam, the student will often review the textbook and notes more frequently than they would have if they studied for a more traditional exam. Additionally, students are more likely to work in group study sessions, summarize material in their own words, and ask questions in class. While the student thinks that she/he is getting a break, in reality, she/he is learning while completing the test and being encouraged to take the work seriously. In a study by Weber et al. (1983), scores on knowledge items were significantly higher on take-home tests, a result attributed partially to the additional time students spent looking up answers.

Take-home tests help increase student knowledge about the information that will be covered in class by providing a base of pre-existing knowledge to which lectures and class activities can attach themselves. Students also have additional time to complete the assessment and therefore, they are not rushing through the test like they may be with an in-class examination, thereby reducing the level of student test anxiety. According to Rich (2011), giving students work to take home can reduce test anxiety, incentivize students to work collaboratively and elicit study habits that are at a deeper level. In his experiment on take-home examinations and retention, students indicated that when they were given tests to complete out of class, they learned more and studied harder.

## Short answer tests

Short answer questions give students a better chance to explain their thinking behind an answer than multiple-choice questions do (Tamir, 1990) and promotes more in-depth studying as students must be able to think conceptually to do well (Balch, 2007). Short answer questions can cover a wider range of content than a multiple choice item, and also allow for the teacher to demand integration of themes and ideas from the students. Short answer questions reduce the possibility of guessing. Further, when grading these examinations, teachers can see or understand the point the student was trying to make, as opposed to multiple choice tests where there is only one right answer. This proposition is supported by research which indicates that more difficult tests promote greater learning than simpler tests (Gay, 2005). In a study by Balch (2007), students who were expecting a short-answer test performed better on definition questions in a multiple choice test than did students expecting a multiple-choice test. Balch suggested that the study practices that students use with short-answer examinations involve elaboration, rather than merely an attempt to recall, which promotes performance on more difficult test questions and deeper understanding of material.

## Formative summative assessments (FSA)

Wininger defined formative summative assessment as “the measurement of student progress before or during instruction for the expressed purpose of modifying instruction and improving student performance by going over exams in class with students and garnering both quantitative and qualitative feedback from the students about their comprehension” (2005, p. 164) Formative summative assessments (FSA) are a way for you and the student to communicate and help them gain a better understanding of the material. FSA's inform both teachers and students about student perception and allow timely adjustments to be made. FSA's are done to improve student understanding and the quality of teaching by providing feedback for both the teacher and the student about learning progress with the goal of improving both instruction and learning (Wininger, 2005). As we are teaching, we can use FSAs to find out how well students comprehend the instruction (Harlen and James, 1997). One example of an FSA is reviewing practice examinations and answering questions about items on which many students are confused, or identifying questions these students may have about the material before the real examination is administered. Some instructors will give practice exams that check on student knowledge, and then use statistical analysis of those practice exams to reiterate or re-explain information that students are finding difficult (Black, 1993).

In an article by Wininger (2005), a teacher examined the use of one FSA method—namely, a review of questions and explanations of correct answers after students had already taken their first examination. In his study the teacher gave two of his classes the same examination. After the examinations were returned he used the FSA method for Class A allowing the students to review and ask questions to help them obtain a better understanding of key concepts covered in the exam, while Class B did not receive any review of the examination. One week later the classes were given the same exam for extra credit to see whether the class who was given the exam review would score higher than the class who did not receive the examination review. In the results, Wininger found that there were no significant differences between the two classes on the initial exam administration. However, students who received the FSA method scored significantly higher on the retake. Students exposed to the FSA method demonstrated an improvement of almost 10% in their test scores, whereas scores for students in the control group improved by only 2%. The results of this study support the effectiveness of the FSA method with regard to student comprehension.

## Audience response systems (ARS)

According to Cain and Robinson (2008, p. 1), “Audience response systems are an increasingly popular tool in higher education for promoting interactivity, gathering feedback, pre-assessing knowledge, and assessing students' understanding of lecture concepts.” Audience response systems (ARS) can give students a chance to evaluate what they have learned and how beneficial they felt each lesson was to them. There is an increased motivation to be engaged in the lesson when students get the chance to participate in ARSs (Doucet et al., 2009). It is important that teachers find ways for students to engage in lessons in order for the students to be able to get a chance to give their feedback on what they were taught. Once given this feedback the teacher can then alter the plan of instruction or students can work out misunderstandings with their peers or classroom discussion. According to an article by Stowell and Nelson (2007), increasing student participation is one of many strategies that could lead to improved student learning. To increase student participation, instructors can use “active student responding” methods. Using clickers, or giving student the ability to text answers to questions through a website like www.pollanywhere.com can help gain more feedback from more students, because their responses will be anonymous (Dallimore et al., 2010).

## Making instruction and assessment responsive to student learning style differences

Some research suggests that helping students and being aware of their learning styles can help them develop better study habits. Teachers can also benefit from information about their students learning styles by incorporating the learning styles of their students into lesson plans (Charkins et al., 1985). This may be done by placing students in learning situations with other students whose learning strengths are different from their own which allows them to practice skills in areas that are opposite to their current strengths (Pashler et al., 2008). As a result, teachers who create multiple forms of assessment to match learning styles may facilitate student performance at their level of competence by removing barriers that uncomfortable test formats can create. Some of the learning styles which have been identified are: auditory (learning best through hearing), visual (learning best through seeing), and kinesthetic (learning best when concepts are more hands-on).

Although most people use a mixture of all three learning styles there is a broad belief among educational researchers that they usually have a clear fondness for one (Kolb, 1984; Leite et al., 2010; see also the implications behind Fleming et al., 2011). Knowing and understanding the types of learning styles is important for students. To find out what your learning style is, you may use an index of learning styles questionnaire like the one at http://www.engr.ncsu.edu/learningstyles/ilsweb.phpl (Soloman and Felder, 1993). Participants will be asked a series of questions to which they will respond. At the end the results of the questionnaire will show which style of learning best fits the participant and which styles fit the least. Once students discover their learning style it can become much easier for studying and less stressful when it comes to homework because students are now aware of what methods of learning are optimal for themselves as individuals (Felder and Silverman, 1988). For example, if you know that you are more of a visual learner, one who prefers graphs and pictures, as opposed to a verbal learner, one who prefers to hear or read information, when looking for directions, you know you are more likely to be successful by looking at a map as opposed to hearing someone give you directions.

Presenting course material that reflects each of the six learning strategies above can help to elicit deeper approaches to learning than standard learning strategies which focus solely on memorization and isolated facts. In particular, the use of learner-centered assessment methods can encourage students to connect new material to previously learned concepts, and/or apply them to real life. For a more in-depth discussion of surface and deep processing, see Chin and Brown (2000).

In conclusion, the use of learner-centered assessment methods can produce more effective instruction, deeper study strategies, and longer-term retention of material than the more traditional methods. Specifically, teachers are encouraged to implement one or more of the strategies discussed in this paper; namely, short quizzes before important readings, take-home examinations, short answer essays, Formative Summative Assessments, student learning style inventories, and ARS.

One way to conceptualize how these strategies might work together would be to first have each student identify his/her learning style, so that the students and their instructor might become knowledgeable about the strengths and weaknesses present in the class. Knowing about the student learning styles that are represented in a class will allow an instructor to create groups that may be more effective, because they have more diverse skillsets. Students can use the knowledge of their strengths and weaknesses to use study strategies which capitalize on the approaches which will lead them to the best outcomes.

After an instructor has gathered the information about learning styles from his/her students, s/he can now engage with the other strategies in this paper in a way that is conversant with that information. Alternate methods for completing homework assignments or assessments can be devised. ARS can be infused into instruction, to allow all students the chance to demonstrate understanding or raise questions. In so doing, the instructor can communicate a genuine interest in student learning, and continually seek improvement in the art of teaching.

### Conflict of interest statement

The authors declare that the research was conducted in the absence of any commercial or financial relationships that could be construed as a potential conflict of interest.
